# Circulating adiponectin and cardiovascular mortality in patients with type 2 diabetes mellitus: evidence of sexual dimorphism

**DOI:** 10.1186/s12933-014-0130-y

**Published:** 2014-09-10

**Authors:** Claudia Menzaghi, Min Xu, Lucia Salvemini, Concetta De Bonis, Giuseppe Palladino, Tao Huang, Massimiliano Copetti, Yan Zheng, Yanping Li, Grazia Fini, Frank B Hu, Simonetta Bacci, Lu Qi, Vincenzo Trischitta

**Affiliations:** Research Unit of Diabetes and Endocrine Diseases IRCCS Casa Sollievo della Sofferenza, Viale Padre Pio, 71013 San Giovanni Rotondo, Italy; Department of Nutrition, Harvard School of Public Health, Boston, MA USA; Shanghai Clinical Center for Endocrine and Metabolic Diseases, Shanghai Institute of Endocrine and Metabolic Diseases, Department of Endocrine and Metabolic Diseases, Ruijin Hospital Affiliated to Shanghai Jiao Tong University School of Medicine, Shanghai, China; Unit of Biostatistics IRCCS Casa Sollievo della Sofferenza, San Giovanni Rotondo, Italy; Department of Medicine, Brigham and Women’s Hospital and Harvard Medical School, Channing Division of Network Medicine, Boston, MA USA; Unit of Endocrinology, IRCCS Casa Sollievo della Sofferenza, San Giovanni Rotondo, Italy; Department of Experimental Medicine, Sapienza University of Rome, Rome, Italy

**Keywords:** Adipokines, Prospective studies, Paradoxical effect, Sex-linked genes

## Abstract

**Background:**

The pathogenesis of cardiovascular (CV) mortality, whose rate is increased in type 2 diabetes, is poorly understood.

While high serum adiponectin is associated with increased CV mortality in the general population, no data are available in type 2 diabetes.

We here investigated whether this counterintuitive association was observable also in diabetic patients and whether it was sex-specific.

**Methods:**

Three prospective cohorts were analyzed: 1) Gargano Heart Study (GHS; 359 patients, 58 events/1,934 person-years; py); 2) Health Professional Follow-up Study (HPFS; 833 men, 146 events/10,024 py); 3) Nurses’ Health Study (NHS; 902 women, 144 events/15,074 py).

**Results:**

In GHS serum adiponectin predicted CV mortality in men (hazard ratio, HR, and 95% CI per standard deviation, SD, increment = 1.54, 1.19-2.01), but not women (HR = 0.98, 0.48-2.01).

Circulating adiponectin predicted CV mortality in men from HPFS (HR = 1.44, 1.21-1.72), but not in women from NHS (HR = 1.08, 0.86-1.35), used as replication samples. In a pooled analysis, HRs were 1.47 (1.27-1.70) in 1,075 men and 1.07 (0.86-1.33) in 1,019 women (p for HRs heterogeneity across sexes = 0.018).

**Conclusions:**

This is the first report showing that high circulating adiponectin predicts increased CV mortality in men, but not in women with type 2 diabetes. Further studies are necessary to unravel the mechanisms through which adiponectin influences CV mortality in a sex-specific manner.

**Electronic supplementary material:**

The online version of this article (doi:10.1186/s12933-014-0130-y) contains supplementary material, which is available to authorized users.

The rate of cardiovascular (CV) mortality is doubled in patients with type 2 diabetes as compared to that of non diabetic individuals [1] and definitively remains their first cause of death [1]. The exact pathogenic mechanisms, underlying such increased risk are poorly understood.

Adiponectin, a 244-amino acid protein secreted by adipocytes has insulin-sensitizing, anti-inflammatory, and endothelial protective effects [2,3] and exerts a protective role in myocardial infarction (MI) [4,5] and coronary heart disease (CHD) [6–8]. Thus, it is surprising that, high adiponectin levels predict increased risk of heart failure (HF) [9,10] as well as CV mortality in the general population and in selected clinical settings [11–21]. A similar unexpected paradoxical association with the risk of CV mortality has been also described in patients with type 1 diabetes [22] and in kidney transplant recipients [23]. Whether this is the case also among patients with type 2 diabetes has never been addressed.

Interestingly, it has been recently reported that in humans there is a sex-specific effect of serum adiponectin in the development of type 2 diabetes [24] and in the progression of chronic kidney disease [25]; also data in mice with genetically induced endothelial dysfunction in which adiponectin exerts its beneficial effect on heart function and remodeling in female but not male are compatible with a sexual dimorphism [26]. While some studies have not reported a sex-specific effect of adiponectin on CHD [27] and cardiovascular events [28], no data are so far available on CV mortality.

In order to investigate whether serum adiponectin plays a role in CV mortality in type 2 diabetes in a sex-specific manner, we analyzed data from over 2,000 diabetic patients of European origin from three independent established cohorts from Italy and the US followed over time for several years.

## Material and methods

### Study populations

#### GHS-prospective design

This study comprises 368 patients with type 2 diabetes (ADA 2003 criteria) and coronary artery disease who were consecutively recruited at the Endocrine Unit of IRCCS “Casa Sollievo della Sofferenza” in San Giovanni Rotondo (Gargano, Center East Coast of Italy) from 2001 to 2008, as recently described [29,30]. All patients had either a stenosis >50% in at least one coronary major vessel at coronary angiography or a previous MI. Follow-up information on outcomes was collected yearly from 2002 to 2011. The only exclusion criterion was the presence of poor life expectancy for non diabetes-related diseases. The end-point was here CV mortality. Confirmation of the event was obtained from death certificates (i.e. according to the international classification of diseases’ codes: 428.1- ninth edition - and I21.0-I21.9, I25.9, I46.9-I50.9, I63.0, I63.9, I70.2- tenth edition).

Clinical data at baseline were obtained from a standardized interview and examination. Smoking habits and history of hypertension, dyslipidemia and MI as well as glucose-lowering treatment were also recorded at time of examination. Data regarding medications were confirmed by review of medical records. Serum total and high-molecular weight (HMW) adiponectin were measured in 359 (98%) participants.

The study was approved by the Institutional Ethic Committee IRCCS “Casa Sollievo della Sofferenza”, San Giovanni Rotondo.

#### Health Professional Follow-up Study (HPFS) and Nurses’ Health Study (NHS)

The HPFS is a prospective cohort study of 51,529 US male health professionals who were 40 – 75 years old at study inception in 1986 [31]. Between 1993 and 1999, 18,159 men provided blood samples. The NHS is a prospective cohort study of 121,700 female registered nurses who were 30 – 55 years old at study inception in 1976 when all of them completed a mailed questionnaire on their medical history and lifestyle [32]. A total of 32,826 women provided blood samples between 1989 and 1990.

In both cohorts, information about health and disease has been collected at baseline and biennially by self- administered questionnaires since inception and renal function was estimated by the simplified MDRD equation [33]. Study individuals in the present analysis from both NHS and HPFS were all patients with type 2 diabetes who provided blood samples, during 1993 and 1994 for HPFS and during 1989 and 1990 for NHS, respectively. We followed these subjects and documented incident CV disease and related death after blood draw. Diabetes cases were defined as self-reported diabetes at baseline confirmed by a validated supplementary questionnaire. For study individuals recruited before the release of the American Diabetes Association criteria in 1997, the National Diabetes Data Group criteria were used to define diabetes [34]. The validity of this method has been confirmed [35]. The American Diabetes Association diagnostic criteria were used for diabetes diagnosis from 1998 onwards [36].

CV deaths were confirmed by review of medical records or autopsy reports with the permission of the next of kin [37].

In the current prospective analysis of plasma total adiponectin levels and CV mortality risk, where follow-up began at the time of blood collection, participants with established CV disease (defined as the occurrence of MI or coronary artery bypass grafting or stroke) before the diagnosis of diabetes were excluded from the present analyses [38,39]. Eight hundred and thirty three diabetic men (146 CV deaths during 14-year follow-up) and 902 diabetic women (144 CV deaths during 20-year follow-up) were included.

The study was approved by the Human Research Committee at the Brigham and Women’s Hospital, Boston, MA, USA, and all participants provided written informed consent.

### Sample collection and measurement of circulating total and HMW adiponectin levels

In the GHS-prospective designs blood samples were collected between 8:00 and 9:00 AM after an overnight fast.

In the NHS and HPFS, blood samples were collected in EDTA blood tubes in the men and heparin blood tubes in the women, chilled, and sent back by prepaid overnight courier. Once in the laboratory, the samples were centrifuged and aliquoted into cryotubes as plasma, buffy coat, and red blood cells. Cryotubes were then stored in liquid nitrogen freezers at −130°C or lower [40].

In the GHS-prospective design, serum total and HMW adiponectin concentrations were measured by ELISA (Alpco, Salem, NH) as previously described [41]. The intra-assay coefficients of variation were 5.4 and 4.9, and 5.0 and 4.8% for total adiponectin and HMW adiponectin, respectively.

In the NHS and HPFS, plasma total adiponectin was measured separately, in two different laboratories, by competitive radioimmunoassay (Linco Research, St. Charles, MO). A similar coefficient of variation ranging 3-4%, was observed in both cohorts [42].

### Statistical methods

Patients’ baseline characteristics were reported as mean ± standard deviation (SD), median (range) and percentages for continuous and categorical variables, respectively.

In all prospective studies, the time variable was defined as the time between the baseline examination and date of the event (CV mortality), or, for subjects who did not experience the event, the date of the last available clinical follow-up. Incidence rates for CV mortality were expressed as the number of events per total number of person-years (py). Univariate and multivariate (taking into account several covariates as described in Results) Cox proportional hazards regression analyses were performed to assess the association between adiponectin values and the event occurrence. Risks were reported as Hazard Ratios (HR) along with 95% CI per SD increase in adiponectin levels and for tertiles of its distribution. The same analyses were carried out in males and females from the GHS-prospective design, separately.

Linearity of association was tested comparing, in terms of AIC (Akaike Information Criterion), a linear and a quadratic model.

As a sensitivity analysis, we performed a competing risks analysis, where the non CV death is the competitor of CV death, using subdistribution hazard ratios estimated through Fine and Gray proportional hazards regression [43].

Meta-analysis of pooled estimates was obtained using the fixed effects meta-analysis model. Between-study heterogeneity was assessed using the Cochran Q test.

In the pooled analysis we had 80% power to detect an HR of 1.25 for 1 SD adiponectin with a Type I Error (alpha) of 0.05 both in men and in women.

A p-value <0.05 was considered as significant. All analyses were performed using SAS Release 9.1.3 (SAS Institute, Cary, NC, USA) and R software (package “cmprsk”).

## Results

### The GHS-prospective design

Baseline clinical features of study participants are summarized in Table 1. During follow-up (5.4 ± 2.5 years), 58 CV deaths occurred, corresponding to an overall annual incidence rate of 3.0% (58 events/1,934 py). Median serum total adiponectin was 4.4 (range: 0.97-21.8) μg/ml. Each SD increment of serum adiponectin was associated with a 30% increase in the risk of CV mortality (HR = 1.30, 95% CI: 1.09-1.56, p = 0.005). The linearity of this association was confirmed by comparing AICs (linear: 632.6 vs. quadratic: 633.5).

**Table 1:** **Baseline clinical characteristics of participants from GHS-prospective design, NHS and HPFS cohorts**

	**GHS-prospective design**		**HPFS**	**NHS**
	**Men (n = 242)**	**Women (n = 117)**	**Men (n = 833)**	**Women (n = 902)**
Age (years)	63.6 ± 8.3	66.1 ± 7.5	63.6 ± 8.6	59.1 ± 7.2
Current Smokers (%)	55 (22.7)	9 (7.7)	56 (6.7)	123 (13.6)
Diabetes duration (years)	12.9 ± 8.8	15.7 ± 9.8	7.8 ± 9.6	11.0 ± 9.3
BMI (kg/m^2^)	29.4 ± 4.3	31.8 ± 5.4	27.8 ± 4.6	29.7 ± 6.3
HbA_1C_ % (mmol/mol)	8.6 ± 1.9 (70 ± 20.8)	8.7 ± 1.8 (72 ± 19.7)	7.3 ± 1.5 (56 ± 16.4)	7.1 ± 1.8 (54 ± 19.7)
Total cholesterol (mg/dl)	175.4 ± 46.0	176.7 ± 45.3	209.6 ± 40.4	229.6 ± 45.4
HDL-cholesterol (mg/dL)	42.2 ± 14.2	46.4 ± 15.0	40.2 ± 11.1	51.7 ± 15.9
Triglycerides (mg/dL)	158.5 ± 98.8	140.3 ± 74.3	191.0 ± 101.7	208.9 ± 152.4
hsCRP (mg/L)	6.7 ± 14.3	4.9 ± 8.3	3.2 + 5.4	7.9 ± 8.8
Anti-diabetic therapy (%)	126 (52.0)	68 (58.1)	603 (72.4)	493 (54.7)
Hypertension (%)	198 (81.8)	107 (91.5)	393 (47.2)	599 (66.4)

Continuous variables were reported as mean ± SD whereas categorical variables as total frequency and percentages.

GHS: Gargano Heart Study; HPFS: Health Professional Follow-up Study: NHS, Nurses’ Health Study.

BMI: body mass index; HbA1c: glycated haemoglobin A1c, hsCRP: high sensitivity C-reactive protein.

This association was independent of baseline clinical features including age, sex, smoking habits, BMI, HbA1c, anti-diabetic therapy, hypertension, total cholesterol, HDL cholesterol, triglycerides and hsCRP (adiponectin SD HR = 1.44, 95% CI: 1.14-1.82; p = 0.002). Duration of diabetes was not included in this model because collinear with age at recruitment.

In contrast, no association was observed with non CV death (23 events; HR = 1.07, 95% CI: 0.73-1.55; p = 0.74); this makes very likely that the association we observed with all-cause mortality (81 events; HR = 1.24, 95% CI: 1.05-1.46; p = 0.01) is mainly driven by that with CV mortality.

Finally, competing risks analysis, in which non CV death was used as competing risk, provided further support to the association between serum adiponectin and CV mortality (HR = 1.24, 95% CI: 1.08-1.43, p = 0.003).

According to our predefined aim, data were then analyzed in men and women, separately. Median (range) total adiponectin were 4.2 (0.97-21.8) and 5.0 (0.67-19.2), in men and women, respectively. The association with CV mortality was clearly evident and statistically significant among men (adiponectin SD HR = 1.41, 95% CI: 1.16-1.70), but not women (adiponectin SD HR = 1.02, 95% CI: 0.63-1.62) (Table 2), thus suggesting differences across sex (p for HR heterogeneity 0.09). The linearity of the association was confirmed by comparing AICs (linear: 417.0 vs. quadratic: 417.8). This association did not significantly change in the adjusted model (Table 2).

**Table 2:** **Risk of cardiovascular mortality by 1 SD of circulating adiponectin levels**

		**Model 1**		**Model 2**	
**Study samples**	**Number of participants/events**	**HR (95% CI)**	**p value**	**HR (95% CI)**	**p value**
**Men**					
GHS	242/42	1.41 (1.16-1.70)	5×10^−4^	1.54 (1.19-2.01)	1×10^−3^
HPFS	833/146	1.26 (1.10-1.45)	1×10^−3^	1.44 (1.21-1.72)	1×10^−4^
*POOLED§*	*1075/188*	*1.31 (1.17-1.47)*	*2×10* ^*−6*^	1.47 (1.27-1.70)*	*2×10* ^*−7*^
**Women**					
GHS	117/16	1.02 (0.63-1.62)	0.82	0.98 (0.48-2.01)	0.96
NHS	902/144	0.91 (0.75-1.09)	0.31	1.08 (0.86-1.35)	0.49
*POOLED* ^§^	*1019/160*	*0.92 (0.78-1.10)*	*0.37*	*1.07 (0.86-1.33)*	*0.53*

GHS, Gargano Heart Study; HPFS, Health Professional Follow-up Study; NHS, Nurses’ Health Study.

Model 1: unadjusted.

Model 2: adjusted for age, sex, smoking habit, BMI, HbA1c, anti-diabetic therapy, hypertension, total cholesterol, HDL cholesterol, triglycerides and hsCRP.

^§^pooled analyses were adjusted for “study sample”.

*p = 0.018 vs. model 2, pooled HR in women.

**Figure 1:**
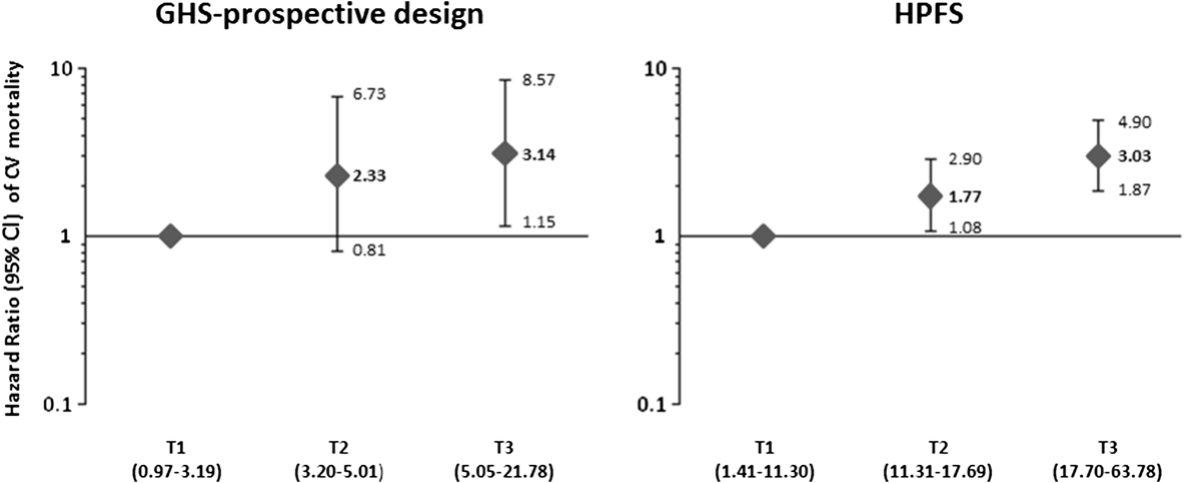
**Hazard ratios (95% CI) of CV mortality in men from the GHS-prospective design and HPFS, according to baseline tertile of circulating adiponectin levels (T1-T3, range in parentheses).** Hazard ratios were estimated by Cox regression after adjusting for age, sex, smoking habits, BMI, HbA1c, anti-diabetic therapy, hypertension, total cholesterol, HDL cholesterol, triglycerides and hsCRP.

When men were stratified according to tertiles of adiponectin levels a trend toward an increased risk of CV mortality from tertile 1 (i.e. the lowest values) to tertile 3 (i.e. the highest values) was observed (adjusted p for trend = 0.026) (Figure 1).

Very similar associations both in the whole sample as well as in men and women analyzed separately were obtained for HMW adiponectin (see Additional file 1: Table S1).

### The HPFS

The baseline clinical features of study participants, including by study design, only male patients, are summarized in Table 1. During an average 14 years (1994–2008) of follow-up since blood samples were obtained, 146 CV deaths occurred in 10,024 py, corresponding to an overall annual incidence rate of 2.8%. Median plasma total adiponectin was 14.07 (range: 1.41-63.78) μg/ml. Mirroring the results obtained among men from the GHS-prospective design, plasma adiponectin levels were associated with CV mortality (adiponectin SD HR = 1.26, 95% CI: 1.10-1.45) (Table 2). The linearity of this association was confirmed by comparing AICs (linear: 1583 vs. quadratic: 1590). This association did not significantly change in the adjusted model (Table 2).

Further adjustments for alcohol consumption and eGFR values (which were not available in the GHS) did not change the observed association (adiponectin SD HR = 1.46, 95% CI: 1.24-1.72).

Also in this case tertiles stratification according to adiponectin levels, revealed a trend toward an increased CV mortality rate (adjusted p for trend <0.0001) (Figure 1).

### The NHS

Baseline clinical features of study participants, including by study design, only female patients, are summarized in Table 1. During an average 20-year follow-up (1990–2010), 144 CV deaths occurred 15,074 py, corresponding to an overall annual incidence rate of 1.9%. Median plasma adiponectin was 5.88 (range: 0.80-42.07) μg/ml. Mirroring the results obtained among women from the GHS-prospective design, total plasma adiponectin was not associated with CV mortality (HR = 0.91, 95% CI: 0.75-1.09) (Table 2). This association did not significantly change in the adjusted model (Table 2). Similar results were obtained after further adjustment for alcohol consumption and renal function (HR = 1.05, 95% CI: 0.85-1.31).

### Pooled analysis

In a pooled analysis, the association between circulating adiponectin and CV mortality was strongly evident among men (adjusted HR = 1.47, 95% CI 1.27-1.70, p = 2×10^−7^), but not among women (adjusted HR = 1.07, 95% CI 0.86-1.33, p = 0.53) Table 2. The difference between HRs obtained in men and women was statistically significant (p for heterogeneity = 0.018).

## Discussion

In this study we investigated in three different cohorts the association of circulating adiponectin on CV mortality in men and women with type 2 diabetes. To the best of our knowledge, this is the first report showing that i) adiponectin is positively related to CV mortality in type 2 diabetes and ii) a sexual dimorphism influences such paradoxical association, which is observable only in men.

Despite the direct association between serum adiponectin and CV mortality is difficult to reconcile with the well-recognized role of adiponectin as an anti inflammatory, anti-atherogenic and insulin sensitizer factor [2,3] and with the previously reported association with reduced risk of CHD [6,7,27], the same counter intuitive finding has been recently described in individuals from the general population and several selected clinical setting [11–17,19–23]. In addition, high adiponectin levels have been reported to predict all-cause mortality in the general population and in patients with CV disease [18–21], as well as in elderly people with type 2 diabetes [44].

Taken together our present and all previous studies strongly point to a paradoxical association of serum adiponectin on mortality rate.

The biology underlying such unexpected association is unknown and cannot be unraveled by observational studies. One can here only speculate that, in our study, increased adiponectin may be interpreted as a marker of a tentative, though not sufficient, homeostatic mechanism in individuals who are at high CV risk [45]. Conversely, it is also possible that in some individuals, serum adiponectin is increased because of adiponectin resistance, which, in turn, does have the potential of playing a direct pathogenic role in increasing risk of CV mortality [3,46,47]. It is also possible that increased adiponectin levels is a consequence of increased N-terminal pro brain natriuretic peptide (NT-proBNP) [18], an established marker of CV disease.

The second important finding of our study is the sex-specific association between circulating adiponectin and CV mortality with the deleterious role of high adiponectin levels being observable only among men. Controversial findings have been so far reported as far as a sexually dimorphic association of adiponectin with metabolic and atherosclerosis-related clinical events is concerned. In fact, while no sex-specific association has been described on CHD in the general population [27] and on CV events in a very small sample of patients with type 2 diabetes [28], high adiponectin levels do predict progressive kidney dysfunction among men, but not women, in individuals with chronic kidney disease [25].

Whether the sex specific associations of adiponectin on metabolic and atherosclerosis-related events we and others [24,25] observed is due to the interaction between circulating adiponectin and either sex-linked genes and/or sexual hormone effects is not known and deserves further investigations. Sex-specific associations have been reported also for other CV risk factors, including those between elevated plasma levels of sTNF-receptors and coronary heart disease [48], between uric acid and atrial fibrillation [49] and between prolactin and cardiac remodeling [50]. A better understanding of the different role of adiponectin on CV disease across sexes, might be gained by verifying whether adiponectin changes, as obtained by interventional studies, are differently related to CV outcomes in males and females.

We acknowledge that the statistical level of sex-specific association indicate the need of caution in interpreting our results. Nonetheless, while waiting for further studies in additional samples, present and previous findings [24,25], definitively suggest the need of taking into account possible sex-specific associations when addressing the role of CV risk factors; in addition, they point to the need of setting up studies specifically designed to unravel the different biology underlying CV risk in men and women.

Our study has some strength as follows. We utilized well-established prospectively analyzed cohorts with a completeness of information, including standardized clinical evaluations and hard end-points validated by medical records or death certificates. In addition, in the GHS-prospective design, very similar data were obtained with HMW-adiponectin, the biological active form of circulating adiponectin, thus reinforcing data obtained by measuring total adiponectin levels. Most importantly, the sex-specific association of adiponectin on CV death, which has been validated in independent samples, is entirely novel and plays the important function of reinforcing the belief that a different biology underlies CV risk in men and women [51]. Finally, since our results were very similar in individuals from both Italy and the US, it is very likely that they can be generalized to all diabetic patients of European ancestry, no matter where they live and the environment they are exposed to. In this context, it is of note that several clinical features, including HbA1c, the proportion of hypertension, and - as far as males from HPFS are concerned - a relatively low proportion of smokers -, were quite heterogeneous across study populations.

A major limitation of our investigation is intrinsic to its very nature. As an observational study, it cannot addresses mechanistic explanations of the association we observed, with only hypothesis being offered.

In addition, it remains unknown whether the paradoxical association between serum adiponectin and CV mortality we observed in men of European ancestry is a general phenomenon also observable in individuals of different ancestries. Also, given that adiponectin measurement was carried out in different specimens (i.e. serum or plasma) by three different laboratories using different assays, a direct comparison of absolute values across the different study samples cannot be done. Finally, the role of potential bias affecting adiponectin levels, including polypharmacy, diabetic kidney disease and other residual or unmeasured confounding factors not taken into account in our study cannot be excluded, thus inviting for caution in interpreting our results.

In conclusion, to the best of our knowledge this is the first study reporting that, quite unexpectedly given its beneficial role in atherosclerotic processes [2,3], high circulating adiponectin predicts increased CV mortality in patients with type 2 diabetes. It is of note that such association was only observed in diabetic men, whereas no association at all was detected in diabetic women. Further studies aimed at unraveling the mechanisms through which adiponectin influences CV mortality and how this occurs in a sex-specific manner are certainly required. The composite, not straightforward, scenario drawn by our findings points to the general need of characterizing in deep details the role of novel and promising markers of CV disease before to upgrade them at the level of usable tools in the clinical set.

## Additional file

Additional file 1: Table S1. HR (95% CI) of cardiovascular mortality by 1 SD of serum HMW adiponectin levels in the GHS-prospective design.
