# Supplementary material for: Circulating adiponectin and cardiovascular mortality in patients with type 2 diabetes mellitus: evidence of sexual dimorphism
Source: Cardiovasc Diabetol. 2014 Sep 10;13:130. doi: 10.1186/s12933-014-0130-y (PMC4172916; doi:10.1186/s12933-014-0130-y)
Supplement: Additional file 1: Table S1. — HR (95% CI) of cardiovascular mortality by 1 SD of serum HMW adiponectin levels in the GHS-prospective design. [file 12933_2014_130_MOESM1_ESM.doc]

**Additional file 1: Table S1. HR (95% CI) of cardiovascular mortality by 1 SD of serum HMW adiponectin levels in the GHS-prospective design.**

| **Whole sample (N=359)** | | **Men (N=242)** | | **Women (N=117)** | |
| --- | --- | --- | --- | --- | --- |
| *Model 1* | *Model 2* | *Model 1* | *Model 2* | *Model 1* | *Model 2* |
| 1.26 (1.05-1.51)  p=0.011 | 1.35 (1.07-1.71)  p=0.011 | 1.40 (1.16-1.66);  p=2.6x10^-4^ | 1.55 (1.20-2.01)  p=0.001 | 0.90 (0.55-1.47);  p=0.866 | 0.87 (0.43-1.77);  p=0.071 |

HMW: high molecular weight; mean±SD = 3.15±2.5 µg/ml

Model 1: unadjusted

Model 2: adjusted for age, sex, smoking habit, BMI, HbA1c, anti-diabetic therapy, hypertension, total cholesterol, HDL cholesterol, triglycerides and hsCRP
